# Self-DNA Sensing by cGAS-STING and TLR9 in Autoimmunity: Is the Cytoskeleton in Control?

**DOI:** 10.3389/fimmu.2021.657344

**Published:** 2021-05-18

**Authors:** Roberto Amadio, Giulia Maria Piperno, Federica Benvenuti

**Affiliations:** ^1^ Cellular Immunology, International Centre for Genetic Engineering and Biotechnology (ICGEB), Trieste, Italy; ^2^ Department of Biomedical Sciences, Venetian Institute of Molecular Medicine, University of Padova, Padova, Italy

**Keywords:** self-DNA, actin cytoskeleton, cGAS-STING pathway, phagocytes, Wiskott-Aldrich syndrome

## Abstract

Modified or misplaced DNA can be recognized as a danger signal by mammalian cells. Activation of cellular responses to DNA has evolved as a defense mechanism to microbial infections, cellular stress, and tissue damage, yet failure to control this mechanism can lead to autoimmune diseases. Several monogenic and multifactorial autoimmune diseases have been associated with type-I interferons and interferon-stimulated genes (ISGs) induced by deregulated recognition of self-DNA. Hence, understanding how cellular mechanism controls the pathogenic responses to self-nucleic acid has important clinical implications. Fine-tuned membrane trafficking and cellular compartmentalization are two major factors that balance activation of DNA sensors and availability of self-DNA ligands. Intracellular transport and organelle architecture are in turn regulated by cytoskeletal dynamics, yet the precise impact of actin remodeling on DNA sensing remains elusive. This review proposes a critical analysis of the established and hypothetical connections between self-DNA recognition and actin dynamics. As a paradigm of this concept, we discuss recent evidence of deregulated self-DNA sensing in the prototypical actin-related primary immune deficiency (Wiskott-Aldrich syndrome). We anticipate a broader impact of actin-dependent processes on tolerance to self-DNA in autoimmune disorders.

## Introduction

TLR9 and cGAS-STING are the two major DNA sensors that have been implicated in recognition of self-DNA and induction of pathogenic IFN-I responses in monogenic and systemic autoimmunity (1–4). TLR9 is localized in endosomes, and it is preferentially expressed in immune cells, such as B cells and phagocytes, where it scrutinizes extracellular material taken up by receptor-mediated endo-phagocytosis. cGAS-STING is ubiquitously distributed in every cell type, patrolling the cytosol for the presence of viral or self-DNA extruded from inner compartments such as mitochondria and nuclei. The activity of TLR9 and cGAS-STING is intimately linked to membrane trafficking. Both TLR9 and STING translocate from a compartment in which they are inactive to a different one where signaling occurs and both need tight control of DNA localization to modulate their activity. Similarly, they share a common pathway for signal extinction in late degradative compartments. One critical and still poorly understood aspect of DNA sensors regulation is their relationship with cytoskeletal structures and the associated actin regulatory proteins. Triggering of TLR9 actively induces actin remodeling as part of the response, impacting signal intensity. Sharing of some of the downstream signaling components suggests that cytoskeletal remodeling may occur as well during activation of cGAS-STING, for instance during autophagy induction, through molecular mediators, though this remains to be defined. On the other hand, actin dynamics support translocation events required for signal modulation and impact the availability of ligands by controlling compartmentalization and accessibility to DNA.

Here, we will discuss the emerging evidence of the role played by cytoskeletal remodeling and actin regulatory proteins during activation of DNA sensors. Inborn errors of immunity caused by defects in actin regulators are beginning to shed light on this connection and will likely boost additional studies to identify the impact of actin dynamics on the balance between immunity and tolerance to self-DNA.

## Self-DNA and Autoimmunity

The concept of immunogenicity of self-DNA is well illustrated by the presence of antibodies reacting to it, which is a hallmark of several systemic autoimmune diseases and especially well characterized in systemic lupus erythematosus (SLE) (5). Later studies established the contribution of endogenous nucleic acids as triggers of innate activation, notably type-I interferon, in SLE and other autoimmune diseases (6, 7). The structural determinants and cellular conditions that promote the immunogenic properties of DNA have been widely investigated and associated with the development of various autoinflammatory and autoimmune conditions (7–9). Monogenic disorders due to deficiencies in DNA metabolizing enzymes provide a further formal demonstration of the immunogenicity of extracellular and intracellular self-DNA. Mutations in the cytosolic DNA degrading enzyme TREX1 are linked to the rare type I interferonopathy called Aicardi-Goutières Syndrome or AGS (10). Human and mouse models lacking extracellular DNAse-I develop a SLE-like phenotype (11, 12). Mouse models and the recent identification of human mutations in lysosomal DNAse-II confirmed the importance of DNA degradation in lysosomes to avoid IFN-I activation (13, 14). DNAse1L3, a DNAse specifically secreted by dendritic cells and macrophages is required to digest circulating chromatin associated with microparticles, and its deficiency is associated with autoinflammation (15, 16). In the following chapters, we will focus on the regulation of the two main receptors implicated in endosomal and cytosolic self-DNA recognition and autoimmunity. The triggering of RNA innate receptors by endogenous molecules is another important pathway to autoimmunity that has been discussed elsewhere (17, 18).

## TLR9 Signaling

The endosomal DNA receptor Toll-like receptor 9 (TLR9) was discovered in 2000 (19) and extensively reviewed in (4, 20). TLR9 is a transmembrane receptor preferentially expressed in immune cells, such phagocytes and B cells. It detects double-stranded DNA (dsDNA) *via* a leucine-rich luminal region and mediates downstream signaling by recruiting MyD88 to its cytosolic C-terminal TIR domain to promote NF-kβ and IRF7 nuclear translocation and cytokines/interferon type I production (21, 22). At the steady state, inactive TLR9 resides in the endoplasmic reticulum (ER). TLR9 trafficking involves exit from the ER assisted by chaperone Unc93b1, transit through the Golgi, and delivery to endolysosomes (23, 24). Upon arrival into the acidic environment of endolysosomes, TLR9 undergoes stepwise processing that makes it competent to bind ligand, multimerize and initiate signaling (25, 26). Collectively these mechanisms restrict signaling to endosomes, preventing ectopic activation and excessive inflammation. TLR9 binds preferentially CpG-rich sequences in DNA of bacterial origin (19, 27), however the receptor can be activated by endogenous self-DNA under specific circumstances. Engulfed self-DNA becomes immunogenic when combined with antimicrobial peptides, chromatin proteins or when release by mitochondria in an oxidized state, as it occurs during extrusion of neutrophil extracellular traps (28–30). These modifications protect DNA from degradation and increase the time of retention in endosomes, facilitating TLR9 activation, and they have been directly linked to the development of autoimmune diseases (31).

## Trafficking and Cytoskeletal Remodeling During TLR9 Signaling

In parallel to activation of inflammatory genes transcription, TLR9 triggering induces rapid cytoskeletal changes through a cross-talk with actin regulatory proteins. Earlier studies had identified a link between TLR signaling and actin remodeling that leads to increased endocytosis in dendritic cells (32). In macrophages, CpG DNA induces actin reorganization and cell spreading by Src mediated phosphorylation of Vav1, an exchange factor for Rac1, and by activation of paxillin (33, 34). Cell spreading, adhesion, and motility induced by the synthetic ligand CpG-B were shown to depend on Src and to occur before acidification of TLR9 in endosomes, suggesting that cytoskeletal remodeling is an early event establishing before full activation of the signaling cascade (35). In pDCs, phosphorylation of IKK and nuclear translocation of IRF-7 to induce interferon production is blunted in DOCK-2 deficient cells. Although the precise mechanism was not elucidated in this case, it was proposed that DOCK-2-mediated Rac1 activation controls IKK-α through NAPDH and generation of oxygen species (36). In line with this report, recent evidence showed that elevation of the B cell adaptor for PI3K (BCAP) in SLE contributes to heightened IFN-I production *via* the DOCK2-Rac1 axis (37). Extracellular matrix stiffness may also control DNA receptor activity. For instance, actin-dependent mechano-transduction *via* Rho-associated coiled-coil kinase (ROCK) was found to dampen TLR9 signaling in macrophages (38). BCR signaling in B cells is intimately linked to actin dynamics. A network of cortical actin and ezrin confines BCR diffusion at the steady state, preventing spontaneous activation. Actin depolymerization by cofilin and ezrin dephosphorylation free BCR mobility inducing assembly of BCR microclusters and signaling (39). Intriguingly, TLR9 signaling was shown to increase cofilin activity inducing BCR mobility and lowering the threshold of BCR receptor activation. The exact mechanism for cofilin activation was not described, yet this study provides an important link between TLR9 signaling and actin dissolution to favor receptor mobility (40). A very interesting extension to this concept is that actin may also control the distribution and mobility of TLR9 in endosomal membranes, preventing collision and spurious activation. Technical challenges inherent to visualization of TLR9 and actin dynamics on endosomes of immune cells prevent direct analysis, yet some of our observations in cells mutated for actin regulators provided some clues in this direction (see below).

A further poorly unexplored area relates to the control of translocation from the ER to signaling endosomes by actin dynamics. In this direction, one report documented that the cytoskeletal protein FODH2, which is linked to SLE predisposition, is critical to retain TLR9 into IRAP^+^ pre-signaling compartments, preventing activation (41, 42). Budding from the ER, transport to the Golgi and post-Golgi trafficking events are all connected to cytoskeletal remodeling in other cellular systems (43–46), therefore the link between actin and TLR9 signaling in immune cells is likely underestimated. This is in part explained by limitations in applying cutting-edge cell biology approaches to primary immune cells.

## cGAS-STING Signaling

The structure and function of the cGAS-STING axis have been extensively reviewed recently (47, 48). Here we will briefly summarize the major features to focus on self-DNA recognition. The cGAS-STING pathway relies on two independent receptors. cGAS is an enzyme with a positively charged surface that recognizes, preferentially, B-form dsDNA to synthesize cGAMP. The newly formed cGAMP molecules bind to STING, an ER-resident protein in the resting state. cGAMP binding induces a conformational change in the inactive dimer of this protein and translocation to the Golgi/ER–Golgi intermediate compartment (ERGIC) where STING oligomerize forming a hub for recruitment of TBK1 and IKK, resulting in IRF3 and NF-kβ-mediated transcription of IFN-I and inflammatory cytokines. cGAMP can also be transferred to bystander cells in a process that involves the transporter Slc19a1, ATP-gated P2X7R channels, and the LRRCA voltage-regulated anion channels (49–51). This signal amplification strategy is extremely powerful in the case of viral infection but may become detrimental during self-DNA recognition. Mechanisms of cGAS negative regulation include low expression at a steady state and sequestration of inactive forms at the plasma membrane and in protected nuclear structures. Outside the scope of the present review, it has to be mentioned that some evidence points to the cytokine-independent output of cGAS-STING activation (52, 53).

cGAS-STING can be triggered by cytosolic self-DNA originated from mitochondria (mtDNA) or nucleus (genomic DNA, gDNA). Oxidized mtDNA spills from damaged mitochondria because of defective mitophagy or formation of VDAC voltage-dependent anion channel or gasdermin-D-dependent pores in the Mt membrane (54–56). Once in the cytosol, oxidized mtDNA counteracts inhibitory mechanisms launching innate activation (57). Nucleoid instability and compromised mitochondrial structure by TFAM deficiency are further examples of aberrant mtDNA release that cause pathogenic TLR9 and cGAS-STING activation (58–60). There is evidence that also genomic DNA can become immunogenic as exemplified in rare genetic disorders. In Ataxia Telangiectasia or Bloom syndrome, the predisposition to genomic instability causes DNA leakage and cGAS/STING activation (61). Mutations in the cytosolic DNA degrading enzyme TREX1 cause Aicardi-Goutières Syndrome or AGS a type I interferonopathy (10). In this group of diseases, and in familiar SLE, gDNA accumulates in the cytosol because of basal S-phase by-products generation or retroelement transposition. Prominent nuclear damage and genomic instability in cancer cells are further examples where the formation of micronuclei or mobilization of transposable genetic elements can feed the cytosol with DNA leading to cGAS activation and IFN-I production (62, 63).

## cGAS-STING Trafficking and the Connection With the Cytoskeleton

Proteins and mechanisms involved in movements of cGAS-STING across intracellular compartments are being increasingly elucidated and illustrate the critical role of trafficking for signal regulation [reviewed in (64)]. STIM1 is an ER-resident Ca2^+^ binding protein implicated in replenishing Ca2^+^ stores at ER-plasma membrane (ER-PM) contact sites. STIM1 was recently reported to interact with STING and to retain it in the ER. Loss of STIM1 in cellular models causes spontaneous IFN-I production and mutations in STIM1 are linked to a severe immune deficiency with autoimmune complications (65, 66). A specular disease mechanism was identified for COPA syndrome, an interferon-mediated disease caused by STING gain of function. COPA interacts with STING and the mutant form inhibits retrograde transport leading to enhanced type-I interferon signaling (67, 68). Another elegant mechanistic insight on the trafficking process of STING has been recently provided by the characterization of STEEP, the “STING ER exit protein”. STEEP was found to interact with STING and to recruit VPS34 to increase the local concentration of phosphatidylinositol 3-phosphate (PtdIns(3)P), facilitating ER membrane curvature, packaging into COPII vesicles and budding. Accordingly, in STEEP mutant cells, STING is retained in the ER, and IFN-I induction is suppressed (69). TMEM203 is a further adaptor that helps STING exit from the ER and regulates its activation (70). Hence, fine-tuned control of STING retention/exit in and from the ER is key to regulate signaling. Intriguingly, it was recently reported that STING controls the accumulation of F-actin *via* WASP and PI3K in B cells, impacting BCR signaling (71). There is a strong rationale to predict more connections between actin and STING trafficking. For instance, translocation of STIM1 to ER-PM contact sites to refill Ca2^+^ stores was shown to be assisted by F-actin assembly (72). Second, membrane phosphoinositides recruited during STING exit from the ER are likely to influence downstream actin regulatory proteins. Moreover, cytoskeletal remodeling at the Golgi provides an interesting area to understand STING regulation (73, 74).

The second outcome of STING signaling, independent of TBK1 and cytokine activation, is induction of autophagy, which helps signal termination by clearance of cytosolic DNA. Mechanistically, cGAMP-induced LC3 lipidation is proposed to be dependent on WIPI2 and ATG5 and ERGIC membranes as a source of LC3 lipidation (52). As widely documented, actin provides an essential scaffold in each step of autophagosomal biogenesis, through a complex balance of Rho GTPAses and downstream nucleation promoting factors (NPFs). Interaction between WASH and components of the membrane trafficking machinery coordinates initial phases of autophagy, such as sorting of ATG9-positive vesicles to early sites of phagophore biogenesis (75). WHAMM orchestrates actin-comet tails generation to sustain phagophore propulsion and invagination (76) and JMY instead shapes autophagosomes by direct interaction with LC3 (77). To note, luminal actin has been spotted by super-resolution microscopy (78), suggesting that polymerization may continue in the lumen of phagosomes. Some evidence suggests that actin may also be important in the late stage of phagosomal closure, as shown by the requirement for Arp2/3 during selective autophagy (79). More recently, Wiskott-Aldrich syndrome protein (WASp) has been linked to the global orchestration of both general and selective autophagy in myeloid cells (80, 81). Even if a detailed mechanism of WASp-autophagy connections remains to be explored, it can be extrapolated that deregulation of actin dynamics (see below) will impact cGAS-STING signaling in phagocytes.

## The Role of Actin in Maintaining Compartmentalization of Self-DNA

Actin dynamics play essential roles in mitochondrial and nuclear stability and therefore impact DNA confinement and prevention of unwanted cGAS-STING triggering. Transient actin polymerization induced by Arp2/3, cortactin, and formins contributes to mitochondria fission and conversely, actin relaxation allows mitochondria fusion (82, 83). In addition, actin flows were recently shown to ensure mitochondria redistribution to daughter cells during mitosis (84), ensuring a proper metabolic balance and mitochondrial turnover. Importantly, mitochondrial disposal by mitophagy is mediated by Arp2/3-dependent actin polymerization downstream of phosphatidylinositol 4,5-bisphosphate (PtdIns(4,5)P_2_) (85). Failure to degrade damaged mitochondria or problems during fission/fusion processes have been directly associated with mtDNA leakage and unwanted inflammation in SLE (54).

A second important level of actin-mediated regulation of DNA sensing is through the control of nuclear integrity and genomic stability (86). WASp and Arp2/3 have been implicated in preserving genomic stability *via* homology-directed repair of DNA double-strand breaks (DSB) during replication stress and by counteracting nuclear deformation (87, 88). On the other hand, genomic instability has been clearly linked to cGAS activation (63). It is thus reasonable to expect an interplay between perturbed actin dynamics, genomic stability, and cGAS activation. In this direction, uncoupling the cytoskeleton from the nuclear envelope in ATR deficient cells was recently shown to cause accumulation of perinuclear cGAS in conditions of mechanical stress (62, 89, 90). The ESCRT machinery involved in endosomal sealing is also recruited at sites of the damaged nuclear envelope for repair (91, 92). Interestingly, cGAS interacts with multiple ESCRT components, suggesting an interplay between cGAS, ESCRTs, and actin, in determining whether nuclear ruptures and DSB can be repaired or an inflammatory response should be triggered. The cross-talk between nuclear integrity, actin polymerization, and excessive responses to self-DNA has emerged as well in neurodegenerative and aging-related diseases. Increased availability of cytosolic self-DNA can occur in laminopathies and increased cytoskeletal stiffness was found to reduce nuclear plasticity, promoting nuclear blebbing and stimulating cGAS-driven senescence in a model of Hutchinson-Gilford progeria (93, 94).

## Crosstalk Between Endosomal and Cytosolic Sensing in Phagocytes

The conventional model of TLR9 activation by ingested DNA in endosomes and cGAS activation by mt/gDNA in the cytosol is challenged in phagocytes. In fact, the two systems may connect in these cells, a process that we propose to call “cross-sensing”. Early evidence already suggested that phagosomal overloading with apoptotic DNA may induce TLR9-independent sensing (95). This was further supported by the observation that lethality in mice deficient for lysosomal DNase-II was rescued by crossing to STING^-/-^ or to cGAS^-/-^, but not TLR9^-/-^ mice (96, 97). More recently, DNA of dying cardiomyocytes engulfed by macrophages was shown to induce cGAS-STING upon transfer from endosomes to the cytosol (98) and DNA delivered to endosomes of monocytes in type-I diabetes patients was observed to trigger STING-dependent inflammation (99). Interestingly, transfer of ingested tumor DNA in the cytosol of dendritic cells is thought to use the same transfer mechanism of cross-presentation, a specialized trafficking route that grants cytosolic access to exogenous antigens (100). Mechanistically, protein partners such as HMGB1 and LL-37, initially identified as enhancers of immunogenicity in TLR9-mediated responses, possess membrane destabilizing properties and promote the endosomal escape of engulfed self-DNA by mechanical disruption (100–104). Besides the cargo mechanical properties, endosomal membrane stability is regulated by cellular factors, including actin dynamics. For instance, endosomal membrane exchange and integrity during maturation are governed by actin nucleators, Rab GTPases, phosphoinositides, and microtubule motors (105–107). In addition, the process of endosomes sealing *via* recruitment of ESCRT (108) may be related to actin remodeling (109). Evidence comes also from neuronal diseases where enlarged endosomes correlate to activation of cytosolic sensing by ingested DNA. For instance, the accumulation of B amyloid fibrils within endosomes was recently shown to combine with self-DNA enhancing its immunogenicity and IFN-I stimulatory activity (110). Although not yet identified, it is interesting to speculate how deregulated cGAS-STING may also be involved in lysosomal storage diseases such as Niemann Pick Disease type C and Gaucher disease, both characterized by defective endosomal segregation, abnormal trafficking and pathogenic inflammation (111). Importantly, our recent data provided direct evidence that innate cells deficient for the actin regulator WASp carry enlarged and immature endosomes that leak ingested DNA in the cytosol (see below). Remarkably, cross-presentation is increased in WASp deficient DCs (112), further suggesting that cross-presentation and cross-sensing may use similar mechanisms and regulation to coordinate antigen processing and innate activation. Conversely, innate signaling may actively increase membrane permeability to potentiate cross-sensing, which is made possible *via* the orchestration of cytoskeletal dynamics. Of note, two recent studies point to the role of cytoskeletal proteins in enhancing endosome to cytosol transfer in cross-presentation (113, 114). In summary, actin dynamics are linked to the integrity of mitochondrial, nuclear, and endosomal membranes and therefore regulate the DNA sensing by ensuring confinement of self-DNA.

## Actin-Related Immunodeficiencies and the Link to Self-DNA Sensing

**Figure 1:**
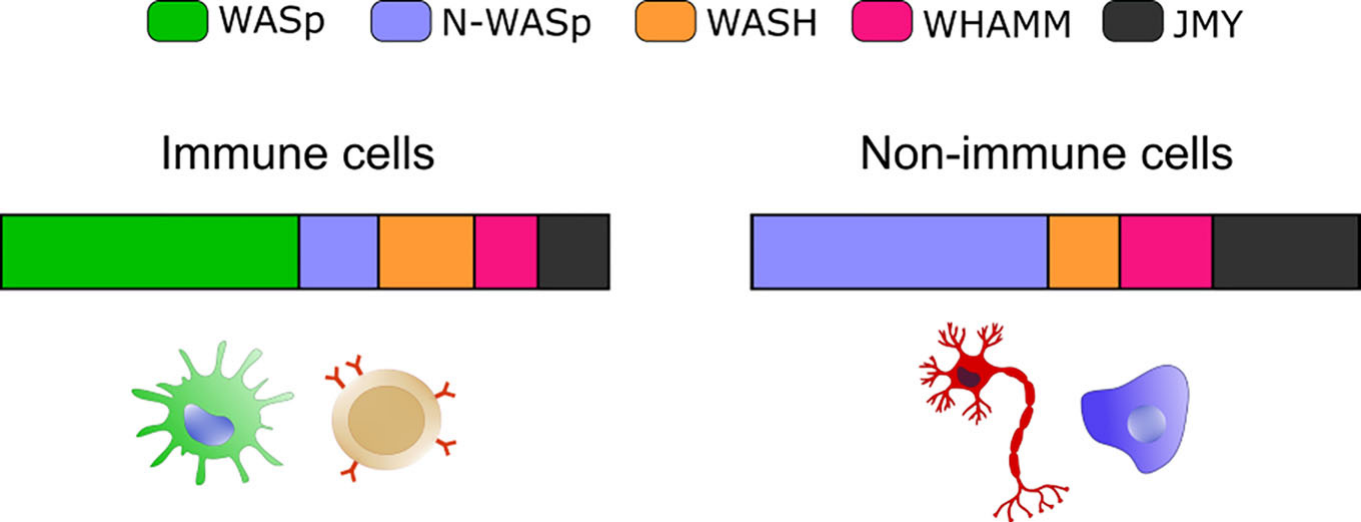
Expression of selected NPFs in immune and non-immune cells. Bar graphs show the relative abundance of the indicated NPF. The “immune cells” plot integrates values from: B cells (bone marrow), Macrophage (bone marrow and spleen) and Classical Monocyte (lung). The “non-immune cells” includes: Epithelial Cell (lung), Fibroblastic Cell (heart), Neuron (brain), Hepatocyte (liver) and Endothelial cell (mammary gland). Plots were obtained by merging expression from different cell types and plotting the resulting average value. Data are from the *Tabula Muris* website (https://tabula-muris.ds.czbiohub.org/) FACS sorted single cell types RNA-seq.

**Figure 2:**
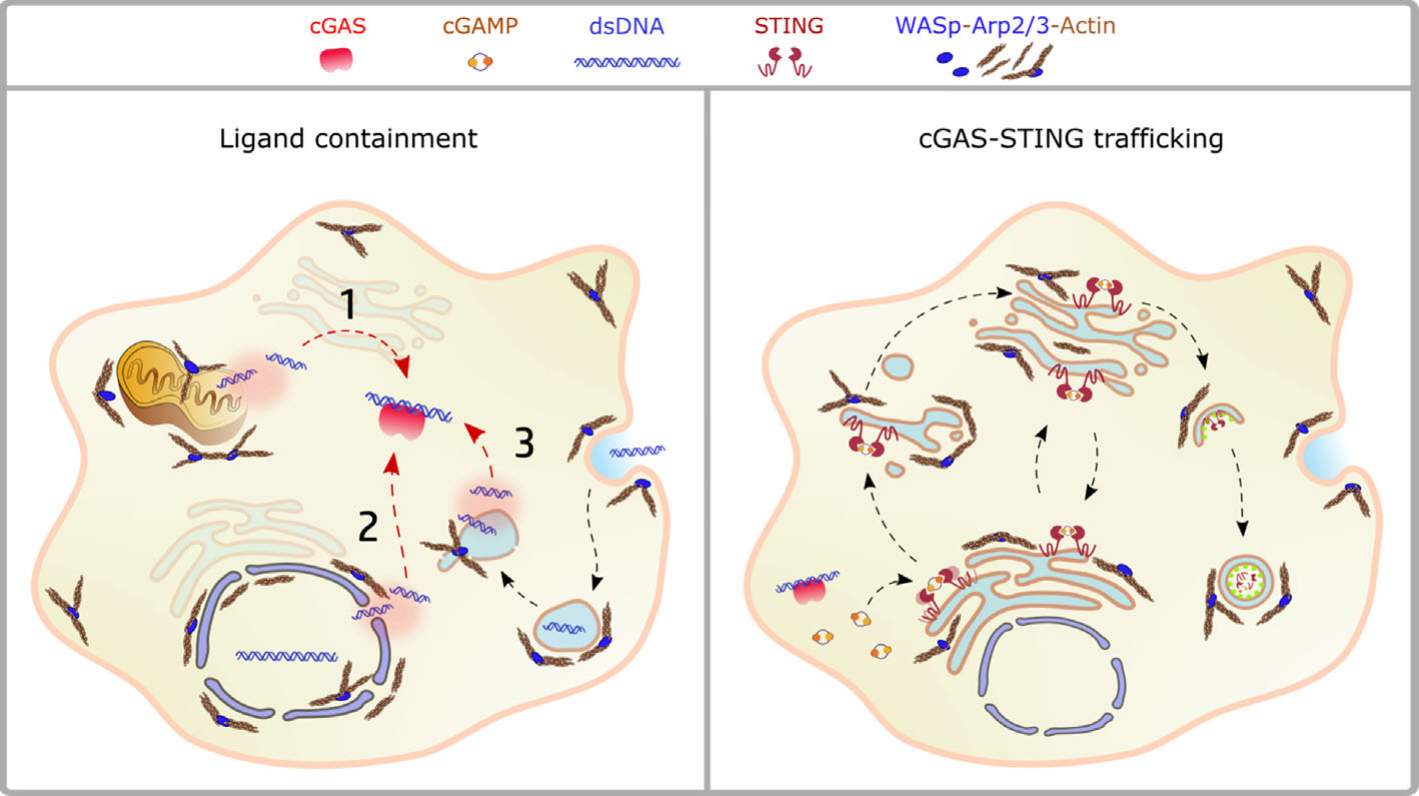
Crosstalk between actin dynamics and cGAS-STING pathway. Left panel: ligand availability into the cytosol may derive from mitochondrial dysfunction (1), nuclear damage (2), endolysosomal leakage (3). The actin cytoskeleton controls both morphology and stability of these compartments, as discussed in the text. Right panel: the scheme depicts STING trafficking steps highlighting the processes controlled by actin dynamics. NPFs of the WASp family regulate vesicles budding from the ER, cargo loading onto COPII vesicles, Golgi stability and post-Golgi trafficking steps, including incorporation into autophagosomes and phagolysosomes.

A plethora of emerging genetic diseases characterized by autoinflammation and immune dysregulation have been associated with cytoskeletal defects and recently reviewed in (115). The recent identification, the limited number of patients, and the lack of appropriated cellular and animal model still preclude precise identification of the driving mechanisms of innate activation, yet their clinical characteristics suggest a possible impaired control of self-DNA recognition that deserve further investigation. Here we will focus on the prototype cytoskeletal-related immune disease, Wiskott-Aldrich syndrome (WAS), the first example of a cytoskeletal-related primary immune deficiency with deregulated type-I interferon driven by cGAS-STING activation. The disease arises from mutations in (WASp), the hematopoietic founder of the WASp family of nucleation promoting factors (NPFs). NPFs of the WASp family include several members that share the ability to activate the Arp2/3 complex and to promote the generation of branched acting fibers on existing actin filaments. WASp activity is regulated by Rho GTPases, SH2-containing tyrosine kinases, Src kinases, and phosphatidyl-inositol (4,5)-bisphosphate (116, 117). NPFs expression levels are highly cell-type specific. While WASp is exclusively present in immune cells that have little to no expression of the other NPFs, non-immune cells express varying the degree of the other NPFs but have no WASp (**Figure 1**). Control of peripheral membrane structures has been attributed to N-WASp, WASp, WAVE, and WHIMP, whereas WASH, JMY, and WHAMM were associated with ER-Golgi and endosome trafficking. Compatibly with its abundance and the poor expression of the other NPFs, WASp was shown to multitask at the cell periphery and intracellularly in immune cells (80, 118). WASp regulates multiple immune cell functions, ranging from BCR and TCR receptor signaling in lymphocytes to migration and phagocytosis in myeloid cells (81). Defects in these processes provide a logical explanation for weak responses to pathogens and increased susceptibility to infections. Somehow paradoxically, the majority of WAS patients also develop a spectrum of autoimmune manifestations with a complex and multifactorial origin, not yet fully elucidated (119). Patients who develop autoantibodies against a wide range of self-antigens, including DNA-associated proteins and mitochondrial antigens, show altered peripheral T-cell tolerance and cell-intrinsic flaws in platelets which promote excessive inflammation and autoreactive B cells activation (120–124). A further emerging key aspect in WAS is the deregulated production of inflammatory cytokines by cells of the innate immune system. Intriguingly, up to 20% of WAS patients still suffer from recurrent inflammatory manifestations even after hematopoietic stem cell transplant (125). In search of a mechanism to link WASp and inflammation, our group uncovered that pDCs in patients and mouse models produce constitutive amounts of type-I IFN and display an enhanced response to stimulation with TLR9 agonists (126). Imaging of individual cells revealed altered trafficking of engulfed multimeric agonists of TLR9, establishing the first direct link between actin dynamics and negative regulation of TLR9 signaling. In addition, WASp null neutrophils are prone to secrete NETs, and their depletion in animal models rescued circulating IFN-I to normal levels, indicating that a combination of cell-intrinsic and cell-extrinsic cues cooperate to exacerbate IFN-I production (127). To further investigate the cellular basis of enhanced IFN-I production we later moved to examine myeloid cells exposed to DNA-immune complexes (DNA-IC), an interferogenic trigger abundant in WAS patients. We found that low doses of DNA-ICs, which are normally tolerated by normal cells, induce IFN-I release in WASp deficient cells. Mechanistically, we found that WASp promotes nucleation of endosomal F-actin and its deficiency causes a delay in endolysosomal maturation, prolonging the transit time of ingested DNA-ICs. Interestingly, stalling in maturation-defective organelles facilitated leakage of DNA-ICs into the cytosol, inducing activation of the STING pathway. Genetic deletion of STING corrected excessive responses of WASp null cells, uncovering cytosolic DNA sensing as a contributor of innate immune dysregulation in WAS (118). Building on this pilot discovery, we hypothesize that WASp may control cGAS-STING mediated IFN-I production by mechanisms beyond endosomal maturation and integrity such as STING trafficking at the ER-Golgi, post-Golgi transfer, and autophagosomal degradation (80, 118) (**Figure 2**). Moreover, by interacting with phosphoinositides (128), WASp may participate in cGAS anchoring at the plasma membrane, a mechanism that prevents recognition of cytosolic self-DNA in myeloid cells (129). Recently it was observed that the formation of actin cages around damaged mitochondria and mitophagy are reduced in WASp null cells (81), suggesting there may be an exaggerated release of mtDNA in the cytosol of WASp deficient cells that could contribute to overall chronic activation of IFN genes. Finally, WASp is also present inside the nucleus. Even if its nuclear function has been uncoupled from actin polymerization, it is tempting to speculate that WASp may limit gDNA leakage and control the epigenetic state of interferon-stimulated genes, for instance in a mechanosensitive fashion. Along this line, it would be interesting to investigate whether, in innate cells, WASp might function as a linker between extracellular matrix stiffness and self-DNA sensing or inflammatory phenotypes.

## Concluding Remarks

The interest in the cGAS-STING system of cytosolic DNA detection has boomed in the last 5 years with an impressive number of reports describing the structural characteristic of cGAS, the range of microbial and endogenous DNA that can bind to it, the trafficking of STING, and the regulatory mechanism ensuring self/non-self discrimination. In hand with basic discovery, the role of cGAS-STING has been appreciated far beyond microbial recognition especially in pathological conditions such as cancer and autoimmune diseases. While the general rules for cGAS/STING functioning have been established, little is known about cell-type- and tissue-specific regulation of DNA sensing. Future studies will be important to discriminate the mechanism of cGAS/STING activation in long-lived, poorly dividing cells as opposed to highly proliferative, short-lived and mobile cells such as immune cells. The coordination of DNA detection by the actin cytoskeleton, a fundamental scaffold that supports most cellular functions, such as endocytosis, intracellular trafficking, and migration, is emerging and will be an important area for future research. The initial finding of enhanced cGAS-STING activation in cells deficient for a prototype regulator of actin dynamics anticipates the importance of this layer of regulation and prompts further analysis in this direction.

## Author Contributions

RA, GP, and FB wrote the articles. RA and GP prepared Figures. All authors contributed to the article and approved the submitted version.

## Funding

This work was supported by Italian Telethon to FB (GGP GGP14281).

## Conflict of Interest

The authors declare that the research was conducted in the absence of any commercial or financial relationships that could be construed as a potential conflict of interest.
